# Ultralow Interlayer Friction of Layered Electride Ca_2_N: A Potential Two-Dimensional Solid Lubricant Material

**DOI:** 10.3390/ma11122462

**Published:** 2018-12-04

**Authors:** Jianjun Wang, Lin Li, Ziting Shen, Peng Guo, Meng Li, Bin Zhao, Lili Fang, Linfeng Yang

**Affiliations:** 1 Computational and Design Center for Low-dimensional Quantum Material, and College of Science, Zhongyuan University of Technology, Zhengzhou 450007, China; szting2817@163.com (Z.S.); goopoo@zut.edu.cn (P.G.); limeng@zut.edu.cn (M.L.); zhaobin@zut.edu.cn (B.Z.); hnzzfll@126.com (L.F.); 13938420289@163.com (L.Y.); 2 Delivery Devices and Connected Solutions, Eli Lilly and Company, Indianapolis, IN 46285, USA; williamnism@gmail.com (L.L.)

**Keywords:** two-dimensional electride, dicalcium nitride (Ca_2_N), ultralow interlayer friction, density functional theory (DFT)

## Abstract

Dispersion-corrected density functional theory (DFT) calculations reveal that the layered electride of dicalcium nitride (Ca_2_N) exhibits stronger interlayer binding interactions but lower interlayer friction behavior than that of traditional layered lubricants weakly bonded by van der Waals (vdW) interactions, such as graphite, *h*-BN, and MoS_2_. These results are attributed to the two-dimensional (2D) homogeneous conduction electrons distribution in the middle of the interlayer space of Ca_2_N, which yields a smooth sliding barrier and hence ultralow friction behavior. The interesting results obtained in this study have not only broadened the scope of 2D solid lubricants but also enriched the physical understanding of ultralow friction mechanism for 2D systems.

## 1. Introduction

The understanding of friction origins and the search for new effective lubricants have been the focus of tribology studies [1]. Layered bulk materials, such as graphite, hexagonal boron nitride (*h*-BN), and molybdenum disulfide (MoS_2_), exhibit excellent lubricating properties and have been used to reduce friction and wear in mechanical systems for a long time [2]. The two-dimensional (2D) strong covalent bonding in plane and the weak van der Waals (vdW) interaction between adjacent layers can effectively explain the lubricating properties of these traditional solid lubricants [2,3]. Surprisingly, these multilayer or even single-layer bulk materials possess excellent frictional characteristics due to their high load carrying capacity and passivating effect, which can serve as protective coating films or nanolubricants and are used in several systems [4,5,6,7,8]. In addition to the homogeneous structure, recent studies have found that heterostructures made by the stacking of different 2D atomic layer crystals, such as graphene/*h*-BN and graphene/MoS_2_, also exhibit unique electronic structures [9,10] and robust interfacial superlubricity [11,12]. The weak vdW interactions and naturally occurring lattice mismatch between two contacting structures is a key ingredient for this superlubricity phenomenon. All above discussions support that layered materials are not only excellent lubricants from the macroscale to the microscale, but also ideal models for investigations of the mechanism of friction.

Electronic structure calculation based on density functional theory (DFT) has become an effective method to study the tribological properties of solid interfaces [13,14,15,16,17,18,19]. The earlier first-principles theory that tried to solve the friction problem was developed by Zhong et al., who studied the stick-slip motion of a single Pd atom over graphite [13]. The model assumes that the potential energy is completely dissipated, but the dissipating process is not considered. Therefore, the maximum-friction model only can obtain major static frictional parameters, such as the maximal potential barrier, friction force, and coefficient of friction (COF). Wolloch et al. further presented a quasistatic model to obtain an approximation of nanofriction based on DFT [14]. In their model, energy dissipation is considered by atomic relaxations. Zilibotti et al. provided a method to calculate the ideal interfacial shear strength by constructing the potential energy surface (PES) that describes the variation of the adhesion energy between two surfaces in contact as a function of their relative lateral position [15]. Recently, Restuccia et al. released a computational protocol to calculate the intrinsic tribological properties of a solid interface from first principles [16], and the high-throughput *ab initio* approach can be employed to easily and effectively calculate friction. Based on these models, DFT calculations have achieved great progress in friction research of solid materials. Cahangirov et al. determined the frictional figures of merit for a pair of layered honeycomb nanostructures by carrying out *ab initio* calculations [17]. They explained the ultralow interlayer friction in the view of critical stiffness. Gao et al. investigated the interlayer sliding potential of multilayer *h*-BN and graphene. They found that interlayer sliding constraints can be employed to tune the contribution of electrostatic interactions and dispersive forces to the sliding energy profile [18]. Reguzzoni et al. investigated the microscopic origin of the increase of friction under load by DFT calculations [19]. They found that pressure-induced charge transfer from the interlayer region toward the near-layer regions is responsible for the increase of friction. More recently, Wolloch et al. constructed a connection between the intrinsic tribological properties and the electronic properties of a solid interface [20]. They showed that the adhesion and frictional forces are dictated by the charge redistribution occurring due to the relative displacements of the two surfaces in contact. All of the abovementioned research works indicated that the electronic structure is a decisive factor in friction, and that DFT calculation can effectively reveal the frictional mechanism.

**Figure 1:**
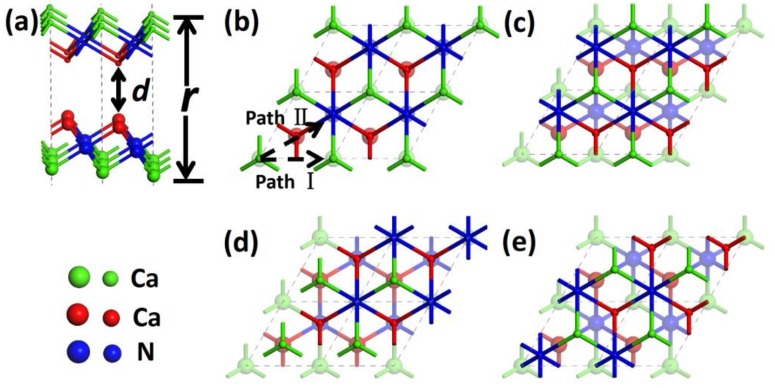
Sliding model. (**a**,**b**) Side and top views of the initial configuration, where *r* and *d* represent the interlayer distance and interfacial space, respectively. As all interfacial Ca atoms from two layers face each other, the configuration was defined as top stacking. The black dotted arrow shows the sliding path of the upper film. (**c**,**e**) Final configurations of the two paths, defined as bridge stackings. (**d**) Another high-symmetric configuration on sliding path II, defined as hollow stacking. The sliding process was modeled by moving the upper layer from the top to the bridge stackings in seven steps with the step lengths of 0.297 and 0.515 Å for paths I and II, respectively. To clearly exhibit the stacking characters, the atoms in different layers are labeled with different sizes.

Recent studies reported a new type of 2D-layered material of dicalcium nitride (Ca_2_N), which has a hexagonal layered structure in the $R\bar{3}m$ space group with high *c/a* ratio, where *a* and *c* are in-plane and out-of-plane unit cell dimensions, respectively [21,22,23,24,25,26]. The layered unit (Ca-N-Ca) is closely packed, and adjacent units have a large separation of about 4 Å (Figure 1a). Electronic structure calculations show that Ca_2_N is a kind of 2D electride with a formula of [Ca_2_N]^+^·e^−^, in which the [Ca_2_N]^+^ unit acts as a positively charged ionic slab and the residual electrons trapped in the interlayer space serve as anions [23,24]. Therefore, Ca_2_N can be regarded as a crystalline structure combined by the Coulomb force between [Ca_2_N]^+^ and e^−^. Experimental and theoretical investigations further found that Ca_2_N exhibits excellent 2D transport characteristics with a high electron mobility (520 cm^2^ V^−1^ S^−1^) and long mean scattering time (0.6 picoseconds) with a mean free path of 0.12 micrometers [23]. The delocalized homogeneous electrons in the 2D interlayer space are responsible for the excellent transport characteristics, which mainly conduct through the 2D space instead of the [Ca_2_N]^+^ layer [23,24]. Lee et al. also calculated the mechanical properties of the structure. They found that the in-plane stiffness is sufficient to support a Ca_2_N monolayer and thus proposed the possibility of obtaining a Ca_2_N monolayer via mechanical exfoliation [24]. Zhao et al. and Guan et al. further investigated the electronic and phononic structures of monolayer and few-layer Ca_2_N, respectively [25,26]. They confirmed that the monolayer and few-layer Ca_2_N samples are stable and have great potential in nanotechnology. It is well known that for a layered structure, larger interlayer distance and high in-plane stiffness are the basic requirements for a 2D solid lubricant [27,28]. Considering that Ca_2_N possesses layered geometry, in-plane mechanical properties, and interlayer distance similar to those of the traditional layered lubricant graphite, excellent friction behaviors are expected. However, the interlayer binding styles are dramatically different for the two kinds of layered materials. Therefore, the special interlayer friction mechanisms of Ca_2_N need to be revealed. However, to our knowledge, there is no experimental or theoretical investigation on the friction properties of Ca_2_N and other alkaline earth subnitrides, such as Sr_2_N and Ba_2_N. Thus, elucidating the tribology properties is important to extend Ca_2_N applications.

In the study, we investigated the interlayer friction properties between two layers of Ca_2_N by applying first-principles calculations. An interesting finding was that the ionic crystal Ca_2_N has comparable friction force with that of the traditional layered lubricants bonded by vdW interactions. The results will contribute to understanding atomic scale friction and extending the range of 2D lubricants.

## 2. Methodology

Density functional theory (DFT) calculations were implemented in the Vienna ab initio Simulation Package (VASP) [29]. The projector-augmented-wave (PAW) method was utilized to model the core electrons and the generalized gradient approximation (GGA), while the Perdew–Burke–Ernzerhof (PBE) functional was employed to describe the exchange and correlation effect [30,31]. The DFT-D2 method was applied to describe the vdW interactions [32], and the default scaling parameter of 0.75 was adopted. A plane-wave cutoff of 600 eV and Monkhorst-Pack grids of 25 × 25 × 1 were used for Brillouin-zone integration [33]. A (1 × 1) surface cell separated by at least a 15-Å vacuum was used to simulate the sliding model. Total energy was converged up to 10^−5^ eV for electronic relaxations. For calculating the band structures, we used Gaussian smearing in combination with a small width of 0.05 eV, and the integration path in the first Brillouin zone was along Γ(0, 0, 0)-Κ(2/3, 1/3, 0)-Μ(1/2, 0, 0)-Γ(0, 0, 0). All internal coordinates were relaxed until the Hellmann–Feynman forces were less than 0.01 eV/Å to optimize geometries. The method of Zhong et al. [13], which has successfully calculated several systems, was employed to calculate the interlayer friction properties of the Ca_2_N system [34,35,36,37]. It should be noted that the method only evaluated the maximum energy barrier caused by variations of the chemical bond strength and the work against an external force along the sliding path, but did not consider the energy dissipation. Therefore, the friction in the study belongs to static friction. 

## 3. Results and Discussion

The calculated in-plane lattice constants and layer thickness of Ca_2_N monolayer are 3.566 and 2.515 Å, respectively. These values are very close to the experimental values of the corresponding bulk structure (3.66 and 2.51 Å) and the other calculation results of the monolayer Ca_2_N (3.562 and 2.516 Å) [21,22,23,24,25,26]. Based on the optimized structure, two sheets of Ca_2_N monolayer were placed to slide against each other along two high symmetrical paths to model the friction process, as shown in Figure 1. Along path I, the two sheets of Ca_2_N alternately encountered the top and bridge stackings. Meanwhile, the top, hollow, and bridge stackings appeared on path II periodically. Therefore, the top, hollow, and bridge stackings are the three key configurations for friction calculations.

**Figure 2:**
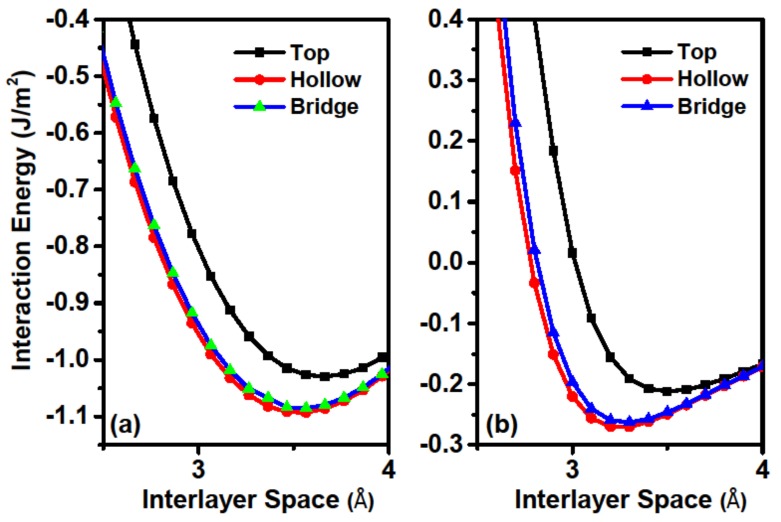
Interaction energy (IE) as a function of interfacial space *d* between two sheets of (**a**) Ca_2_N and (**b**) graphene, respectively. It should be noticed that the two parts have different y-axis scales.

As friction is closely related to the interfacial interaction, we firstly calculated the interaction energy (IE) of the sliding system. IE is defined as the energy of the combined system minus the energy sum of the component films, and was calculated by using the formula $IE=E^{AB}(r)-E^{A}-E^{B}$, where $E^{AB}(r)$ is the total energy of the two contacting films at the distance of *r* and $E^{A}(E^{B})$ is the energy of the separate film. The vertical distance between the bottom and top Ca layers is defined as *r*, as shown in Figure 1a. The load effect was applied by setting different *r* values. For each interlayer distance *r*, only the Ca atoms in the bottom layer of the lower slab and the topmost layer of the upper slab were kept frozen, whereas all other atoms were relaxed in all of our calculations. According to the definition of IE, a more negative IE indicates better stability. The calculated IEs as a function of *r* for top, bridge, and hollow stackings are shown in Figure 2a. For comparison, the IEs for the bilayer graphene system are also presented in Figure 2b. Comparisons of IEs in different stackings reveal that both systems have the weakest interaction at the top stacking, followed by the bridge one, while the hollow stacking has the strongest values. This behavior occurs because interfacial Ca atoms exhibit large spaces for movement and avoid repulsive forces at both the hollow and bridge stackings. These results indicate that the hollow stacking has the largest binding energy (the absolute value of the minimum IE) and is the most stable stacking configuration. To reveal the difference of interlayer binding style between the two systems, we compared the IEs for the two systems. The most striking difference between the two systems is that the IEs for the Ca_2_N system (approximately 1 J/m^2^) is about five times larger than that of the graphene systems (typical physical adsorption of about 0.1 J/m^2^). Therefore, the chemical interlayer bonding character of the Ca_2_N system is dramatically different than the vdW interaction within the graphite system [34]. We next considered the relative difference of IEs between the top and hollow stackings for the two systems. The IE difference at the equilibrium adsorption position is about 0.063 J/m^2^ for Ca_2_N system, which is comparable with that of the graphene system (about 0.058 J/m^2^). We also examined the influence of vdW on the IE in Ca_2_N, and found that the IE difference is virtually unchanged if PBE is used without vdW corrections. These results illustrate that although the two systems have different bonding energies, the difference of binding energy among different stackings is comparable. Interfacial space *d* (as shown in Figure 1a) is another important value for friction calculations, and the calculated values are 3.57 Å and 3.25 Å for Ca_2_N and graphene, respectively. From the comparisons we can see that the Ca_2_N system has a larger interfacial space than that of the graphene system. Therefore, excellent friction behaviors are expected for the Ca_2_N system in the view of its IE and interfacial space.

**Figure 3:**
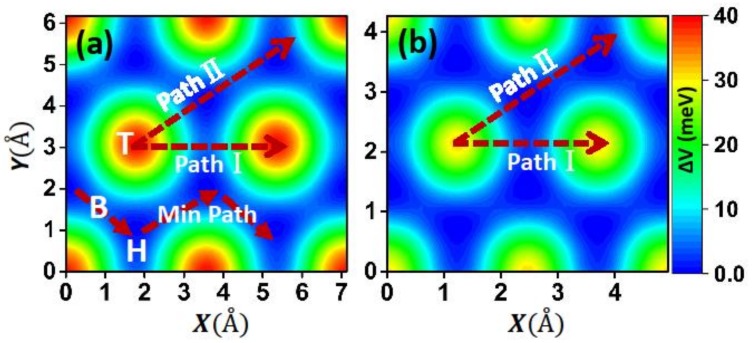
Potential energy surface (PES) for the (**a**) Ca2N and (**b**) graphene systems. T, B, H are the top, bridge, and hollow sites indicated in Figure 1. We also show the two chosen paths and the minimum energy sliding path.

To gain an overall view of friction, we also constructed the static PES by calculating the interlayer IE for different relative lateral positions of the two films at the equilibrium distance [14], as shown in Figure 3. From the figure we can see that the two systems exhibit a similar pattern of PES, with the maximum at the top and minimum at the hollow sites. The maximum is about 40 meV for the Ca_2_N system, which is 1.6 times larger than that of the graphene system (26 meV). However, the length of the sliding path and cell area of the Ca_2_N system are 1.5 and 2 times larger than those of the graphene system, respectively. Therefore, the difference of the lateral force ($f_{\alpha }=-\nabla_{\alpha }v$ experienced by a surface unit cell during its displacement along the direction α) between the two systems should be subtler. We also presented the lowest energy sliding path, as shown in Figure 3a, and the minimum barrier is only about 4 meV, which is comparable to that of the graphene system (about 3.2 meV). This indicates that Ca_2_N could be considered a candidate for lubricant materials. 

**Figure 4:**
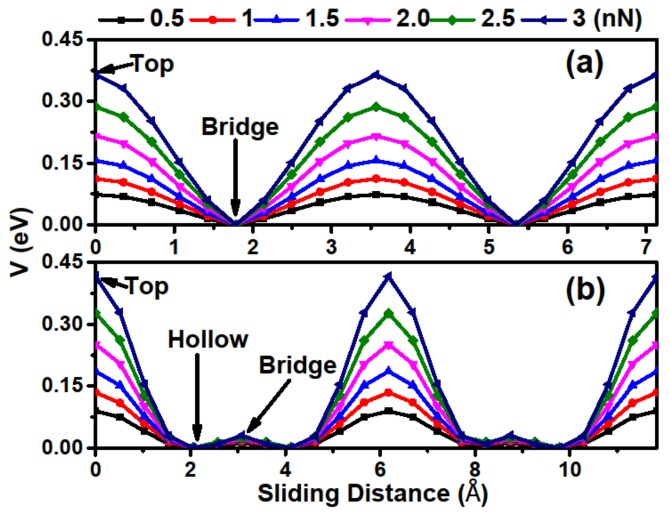
Potential barrier *V* curves along the sliding direction under different loads. (**a**,**b**) *V* curves of paths I and II, respectively.

We next investigated the detailed frictional properties along the two chosen directions by Zhong’s method [13], and more details of the calculating process can be found in our previous works [34,35,36]. Figure 4 exhibits the potential barrier *V* along the sliding path, in which the hollow stacking at path I and the bridge stacking at path II are set to 0 eV. From the figure, one can see that the relative *V* curves periodically fluctuate along the sliding directions, with a maximum at the top stacking and a minimum at the bridge and hollow stackings for paths I and II, respectively. We also considered the load effect, which can be obtained by differentiating the fitted interaction energy function with respect to the interlayer distance [34]. It was found that *V* increases and its curve becomes steeper with increasing loads, indicating that relative sliding is difficult to achieve under high loads. Another important characteristic is that the disparity of the potential barrier amplitude between two paths is not obvious, suggesting that Ca_2_N has an isotropous mean interlayer friction behavior.

**Figure 5:**
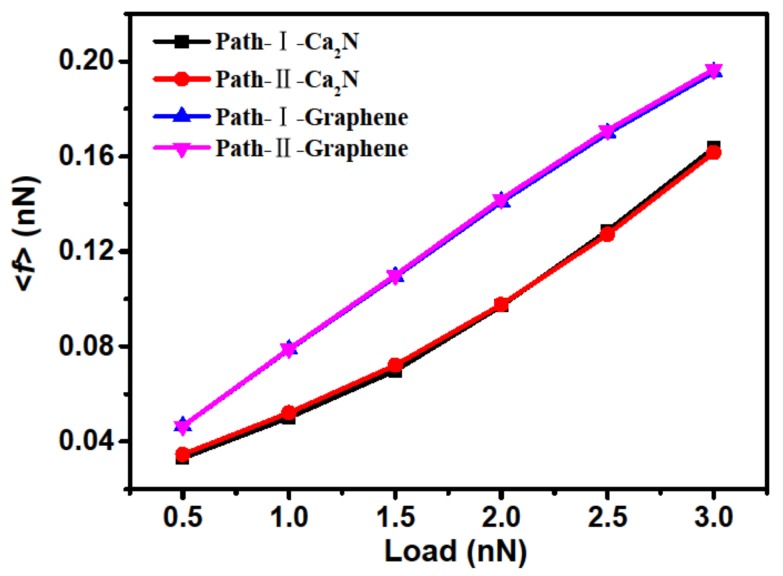
Comparison of mean friction force <*f*> between Ca_2_N and graphene systems.

The mean friction force <*f*> was calculated by dividing the maximum potential barrier by the distance between the two nearest neighbor maximum energies in the sliding direction, as shown in Figure 5. First of all, the curves of <*f*> for both paths are almost coincident throughout the calculated normal forces, which further illustrates that the interfacial friction is isotropous for the Ca_2_N system. By comparison, we also calculated the interfacial <*f*> of the bilayer graphene system. From the comparisons of <*f*> in Figure 5, it can be seen that the Ca_2_N system has lower friction than the graphene system for all chosen loads, which indicates that layered materials with stronger binding can have lower friction, and that friction is closely related to the IEs difference for different stackings but not the absolute IE. It should be noticed that Zhong’s method can successfully deal with the vdW binding layered materials, but it underestimates frictional forces in metallic systems [14]. It is doubtful whether this method is suitable for the calculation of larger adsorption layered systems. However, as the constructed PBS of the Ca_2_N system has the same tendency as the friction calculation by Zhong’s method, the calculated results here are reliable. It also should be emphasized that, although these results reveal static friction at 0 K, and the effects of velocity and temperature must affect the quantitative estimate, such effects will not break the significant ground state mechanism of atomic-level friction. Therefore, bilayer or multilayer Ca_2_N systems may be promising candidates for nanolubricant or coating materials. Additionally, first-principles calculations have also been employed to predict two other 2D electride alkaline earth subnitrides, Sr_2_N and Ba_2_N [21,22], and it was found that they are stable down to monolayer thickness [23]. As these materials have structures similar to that of Ca_2_N, we can infer that this class of materials has excellent friction properties. 

**Figure 6:**
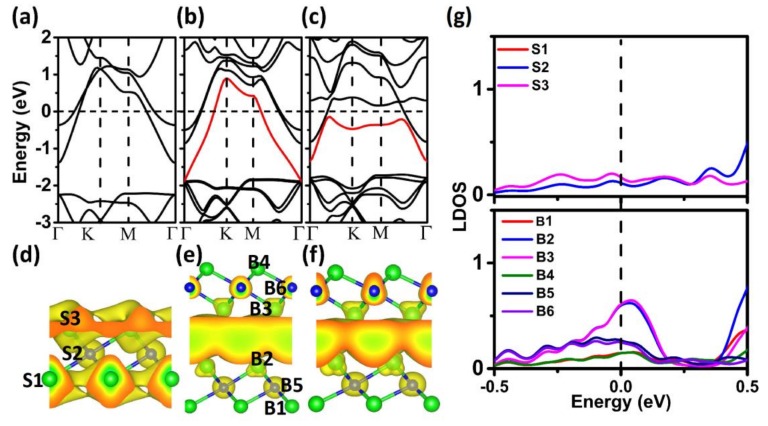
Band structures of (**a**) monolayer Ca_2_N and (**b**,**c**) bilayer Ca_2_N under the loads of 0 and 3 nN, respectively. Fermi energy is set at zero. (**d**) Partial electron density isosurface (with the threshold value of 0.002/Å^3^) for all states within the energy range |E−Ef|< 0.05 eV of (a); (**e**,**f**) for the red bands of (b) and (c), respectively. (**g**) Local density of states LDOS for monolayer (top panel) and bilayer (bottom panel) Ca_2_N. The corresponding atoms are labeled in (d) and (e).

The vdW interlayer interaction is the main mechanism of traditional solid lubricants. Due to the different interlayer bonding styles, the ultralow friction mechanism of the Ca_2_N system may be fundamentally different from that of the vdW layered lubricants. To reveal the ultralow friction mechanism, we calculated the electronic structures of the Ca_2_N system, as shown in Figure 6. The monolayer Ca_2_N is metallic with two bands crossing the Fermi level (Figure 6a), and the corresponding partial electron density shows that the two bands are from the 2D-confined electron layers residing at the two sides of [Ca_2_N]^+^ (Figure 6d). However, when the two sheets are joined, an additional band induced by the coupling effect between the two layers appears, which is mainly from the 2D electron layers confined in the interlayer regions (Figure 6b). As the additional band also crosses the Fermi level, the electrons confined in the interlayer regions are loosely bound conduction electrons, suggesting that the delocalized 2D charge is distributed uniformly in the interlayer space of the Ca_2_N system (Figure 6d), which yields similar IEs at different stackings and smooth *V*. To obtain more information about charge distribution, we plotted the local density of states (LDOS) for monolayer and bilayer Ca_2_N samples. Compared to the monolayer system, there is a clear peak at the Fermi level in the bilayer system, which comes from the two Ca atoms at the interface (B2 and B3 in Figure 6e). These results indicate that electrons confined in the interlayer regions are loosely bound conduction electrons, which is consistent with the above charge redistribution. Therefore, uniform interfacial charge distribution is the main cause of the ultralow friction mechanism of the layered Ca_2_N. For a larger load, the additional band becomes flat and drops below the Fermi level (Figure 6c,f). These results indicate that the confined effect becomes stronger and the homogeneity of the interfacial charge distribution decreases with the increase of the load, which can explain the larger *V* and friction under the larger load. It should be noted that our results are in contrast to the findings of Wolloch et al., as larger adhesion corresponds to larger interlayer friction [20]. This controversy may be attributed to the unique geometric and electronic structures of the Ca_2_N system. The large interfacial space, strong adsorption, and unique 2D uniform interfacial charge distribution make it dramatically different from any other material in their calculations [20].

## 4. Conclusions

In summary, we have investigated the interfacial static friction properties of layered electride Ca_2_N using the vdW-corrected first-principles approach within DFT. Our calculations reveal that the system exhibits strong interlayer Coulomb attractive forces, but an ultralow friction behavior. The findings are fundamentally different from conventional layered lubricant materials, such as graphite, *h*-BN, and MoS_2_, where the interlayer bonding is decided by the relative weak vdW interactions. We used electronic structures to explain the ultralow interfacial friction of Ca_2_N. The uniform interfacial charge distribution brings about a small energy barrier along the sliding path and the easily cleaved nature of Ca_2_N. Because of the large interlayer adsorption, Ca_2_N will not fall off easily when it is used as lubricant, so it can be considered as a kind of green, anti-wear lubrication material. Our results thus identify a class of promising 2D nanolubricants and enrich the physical understanding of friction mechanisms.
